# Endogenous Human Brain Dynamics Recover Slowly Following Cognitive Effort

**DOI:** 10.1371/journal.pone.0006626

**Published:** 2009-08-14

**Authors:** Anna Barnes, Edward T. Bullmore, John Suckling

**Affiliations:** Brain Mapping Unit, Behavioural & Clinical Neurosciences Institute, Department of Psychiatry, University of Cambridge, Cambridge, United Kingdom; Freie Universitaet Berlin, Germany

## Abstract

**Background:**

In functional magnetic resonance imaging, the brain's response to experimental manipulation is almost always assumed to be independent of endogenous oscillations. To test this, we addressed the possible interaction between cognitive task performance and endogenous fMRI oscillations in an experiment designed to answer two questions: 1) Does performance of a cognitively effortful task significantly change fractal scaling properties of fMRI time series compared to their values before task performance? 2) If so, can we relate the extent of task-related perturbation to the difficulty of the task?

**Methodology/Principal Findings:**

Using a novel continuous acquisition “rest-task-rest” design, we found that endogenous dynamics tended to recover their pre-task parameter values relatively slowly, over the course of several minutes, following completion of one of two versions of the *n*-back working memory task and that the rate of recovery was slower following completion of the more demanding (*n* = 2) version of the task.

**Conclusion/Significance:**

This result supports the model that endogenous low frequency oscillatory dynamics are relevant to the brain's response to exogenous stimulation. Moreover, it suggests that large-scale neurocognitive systems measured using fMRI, like the heart and other physiological systems subjected to external demands for enhanced performance, can take a considerable period of time to return to a stable baseline state.

## Introduction

The dominant experimental paradigm in functional magnetic resonance imaging (fMRI) assumes that the brain's response to an experimentally controlled task is independent of its endogenous or background oscillatory activity. In other words, most fMRI experiments are predicated on reflexive rather than adaptive models of brain function [1]. This core assumption is reflected in many ways, including the design of experimental sessions and the use of linear models for time series analysis.

Recent studies have focused attention on the properties of the low frequency (<0.5 Hz) endogenous oscillations that can be measured using fMRI while participants lie quietly in the scanner – at “rest”, at least in so far as they are undisturbed by any other experimental condition. It has been shown that low frequency fMRI oscillations have fractal scaling properties, in common with the electrocardiogram (ECG) and other physiological time series, that can be measured using the Hurst exponent and are modulated by normal ageing, Alzheimer's disease and anticholinergic drug treatment [2], [3]. It has also been shown that the spectral properties or coherence of endogenous fMRI dynamics can be altered during task performance [4] or in the period immediately following completion of a cognitive task [5].

Here we addressed the possible interaction between cognitive task performance and endogenous fMRI oscillations in an experiment designed to answer two questions: 1) Does performance of a cognitively effortful task significantly change fractal scaling properties of fMRI time series compared to their values before task performance? 2) If so, can we relate the extent of task-related perturbation to the difficulty of the task and can we estimate how long it takes after task completion for the brain's endogenous activity to recover or return to pre-task values?

**Figure 1:**
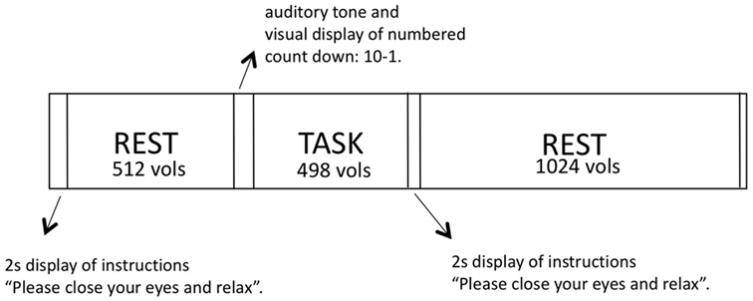
Summary of experimental paradigm.

**Figure 2:**
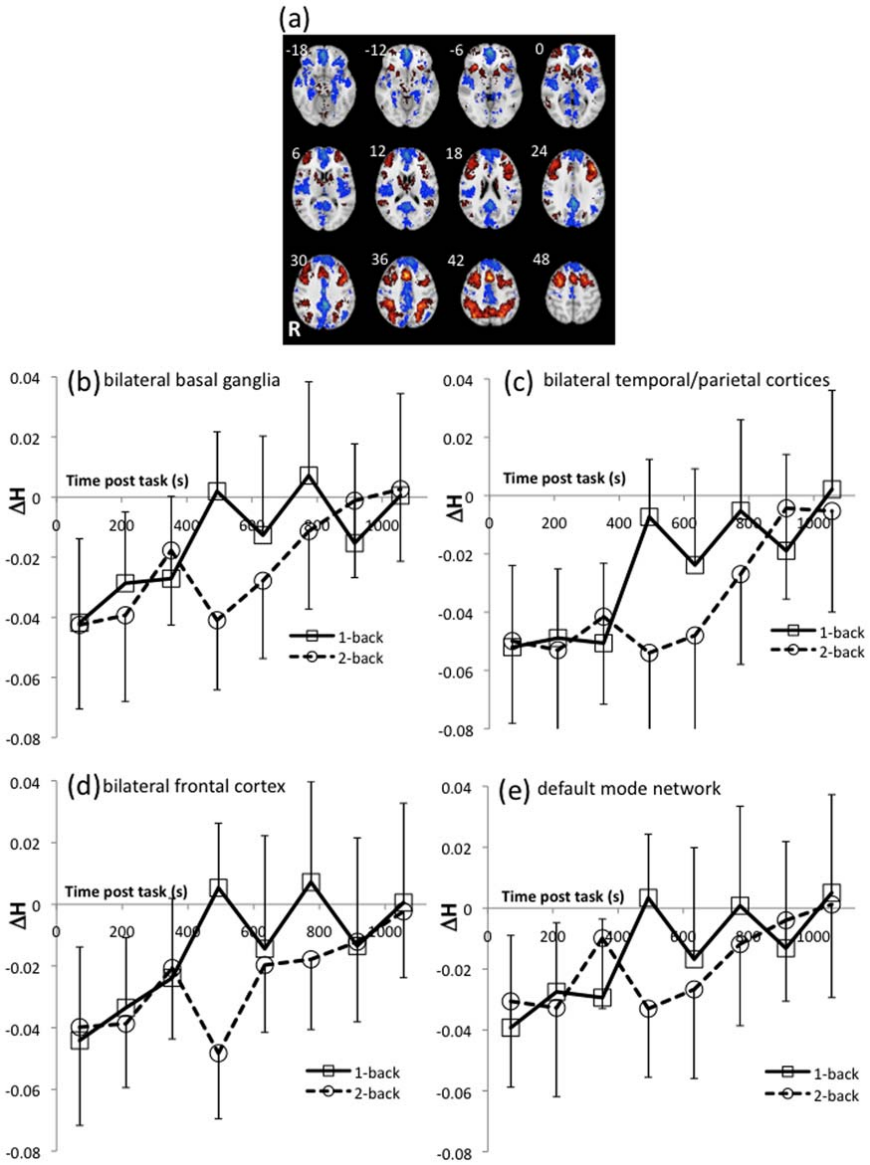
Task-activated brain regions and the recovery of fractal scaling of endogenous oscillations after task performance. (a) Within-group map of activated (red) and deactivated (blue) regions from a contrast of *n*-back versus zero-back (control) trials of the working memory task. Axial slice locations are in mm coordinates of the MNI stereotaxic template. The left of the image is the right of the brain. Threshold for significance was at the cluster level and set such that one false-positive cluster was expected under the null hypothesis (equivalent p = 3.6×10^−3^). (b–e) Post-task recovery of fractal scaling (Δ*H*) for low and high working memory loads, extracted from activated regions clusters 1–3 and deactivated regions cluster 1 (see table 1 for anatomical description). Error bars are between-subject standard deviations. Note that in the immediate post-task period, values of *H* were lower than before task performance, indicating a relative loss of long-range autocorrelations or long memory properties in the endogenous dynamics. Endogenous dynamics tended to recover their pre-task parameter values quite slowly over the course of several minutes following completion of the task and the rate of recovery was faster following completion of the less demanding version of the working memory task.

Fourteen healthy adult volunteers consented to participate in the experiment. Each participant was scanned in two sessions (separated by at least 30 mins) and in each session fMRI data were acquired continuously during a novel continuous acquisition “rest-task-rest” paradigm: during the first resting stage (duration: 9 mins 23 s) participants lay quietly in the scanner; during the task stage (duration: 9 mins 23 s) participants were presented with a blocked periodic *n*-back working memory test [6]; during the second resting stage (duration: 18 mins 46 s) participants were scanned while lying quietly. In total, 2048 three-dimensional images of the brain were contiguously acquired in each session with a sampling interval of 1100 ms (37 mins 32 s overall). The only difference between sessions was the level of difficulty for the working memory test, which was either low load (*n* = 1) or high load (*n* = 2); the order of these different versions was counterbalanced across participants. See Figure 1 for summary of overall experimental design. Brain regions activated or deactivated by task performance on average over all participants were identified by linear modelling and nonparametric inference [7]; Figure 2a and Table 1. Fractal scaling of the pre- and post-task resting time series in activated and deactivated regions was estimated for contiguous segments of 128 time-points by a wavelet-based maximum likelihood estimator of the Hurst exponent (*H*) [2]. Values of *H* in each segment were normalised by subtraction of the value estimated in the segment immediately prior to task. This procedure allowed us to track any changes in fractal scaling, Δ*H*, of endogenous fMRI oscillations over time before and after task performance.

**Table 1:** Brain regions activated or deactivated by working memory task performance.

Anatomical label	Maximal F statistic	Stereotactic co-ordinates of maximal F statistic (mm)
**Activations**		
*Basal ganglia*	10.18	−16, 12, −2
L basal ganglia	10.18	−16, 12, −2
R basal ganglia	8.044	18, 16, 2
*Temporo-parietal Cortex*	22.49	36, −42, 42
L superior temporal gyrus	8.76	−46, −46, 16
R superior temporal gyrus	12.35	56, 50, 20
L inferior parietal lobe	18.7	−32, −46, 38
R inferior parietal lobe	22.49	36, −42, 42
*Frontal Cortex*	29.89	28, 6, 56
L middle frontal Lobe	19.52	−44, 30, 26
R middle frontal lobe	20.89	42, 42, 24
L/R supplementary motor area	26.03	0, 14, 46
**Deactivations**		
*Default Mode Network*	32.59	0, 54, −6
L/R orbito frontal lobe	32.59	0, 54, −6
L/R posterior cingulate gyrus	27.1	−2, −48, 26
L medial temporal lobe	18.09	−28, 10, −22
R medial temporal lobe	14.00	28, 8, −20

Anatomical locations of maximal test statistics are specified in {x,y,z} coordinates (mm) in the stereotactic system of the MNI template image and the number of supra-threshold voxels comprising the 4 most significant clusters designated as activated regions and deactivated regions.

## Results

### Behavioral data

Group mean (±standard deviation) accuracy and reaction time (RT) for the different levels of task difficulty were: zero-back 93.46%±8.00, 0.66 s±0.13 s; one-back 86.92%±10.64, 0.52 s±0.17; two-back 73.12%±17.23, 0.62 s±0.25. There was no significant difference for mean RT between one-back and two-back working memory tasks (t(13) = 1.67, p = 0.06), although there was a significant deterioration of accuracy (t(13) = 2.71, p = 9.36×10^−3^) at higher load.

### Working memory task activation

Figure 2a show regions of significant group response to the contrasts of one- and two-back against zero-back (cluster-level test, equivalent p = 3.571×10^−3^). Activated regions include bilateral superior lateral frontal and parietal regions, supplementary motor area, bilateral head of caudate and anterior putamen, matching the network previously reported in the literature [8]. Areas of relative deactivation include bilateral medial temporal lobes, orbito-frontal cortex, medial frontal cortex, and posterior cingulate that together constitute the so-called default mode network [1], [9].

### Regional mean estimates of the Hurst exponent

The post-task time-course of Δ*H* estimated from the eight, 128-timepoint samples and the corresponding between-subject standard deviations, extracted from the activated and deactivated clusters listed in Table 1 are shown in Figure 2. The regions were defined as the extent of the three-dimensional clusters of suprathreshold voxels in the areas of significant group response (figure 2a). A two-way repeated measures factorial analysis with within-subject factors of working memory load (high [*n* = 2] and low [*n* = 1]), and time (8 post-task samples) was performed within each cluster.

There was a significant main effect of time for all activated and deactivated regions (F(7,91) = 3.65, p<0.002), indicating that fractal scaling of endogenous dynamics changed over the course of several minutes following task completion. The main effect of load was not significant but, importantly, the load-by-time interaction was significant for activated regions of temporo-parietal cortex (F(7,91) = 3.18, p = 0.005) and for deactivated regions of the default mode network (F(7,91) = 2.38, p = 0.028). However, the three-way load-by-time-by-region interaction was not significant (F(7,21) = 1.028, p = 0.43).

Testing the two time points immediately prior to the task yielded a significant effect of time (F(1, 13) = 6.969, p = 0.020), but no main effect of load (F(1,13 = 0.238, p = 0.634) or load-by-time interaction (F(1, 13) = 0.072, p = 0.793).

In order to investigate whether performance was related to the degree of change seen in their fractal scaling, Δ*H* for each participant and each region was regressed against their mean RT and accuracy during performance of the corresponding working memory task. No significant correlations were observed at a threshold of p<0.05. Additionally, the response estimates during the task in each of the regions were also not significantly correlated (p>0.05) with the corresponding Δ*H*.

## Discussion

These results plainly refute the reflexive assumption that endogenous oscillatory dynamics are independent of the brain's response to exogenous stimulation. Moreover, they suggest that large-scale neurocognitive systems measured using fMRI, like the heart and other physiological systems subjected to external demands for enhanced performance, can take a considerable period of time (in the order of 6 minutes) to return to a stable baseline state. This analogy between the brain's response to a cognitively effortful test and the heart's response to an exercise stress test [10] is compatible with the observation that the recovery time for fractal dynamics of fMRI time series was greater following performance of the more effortful version of the working memory test.

FMRI time series with a 1/*f*-like power spectrum are well modelled as fractional Gaussian noise with signal variance, *σ*, and a power law scaling function indexed by a single (Hurst) exponent, *H*, where the fractal dimension *D* = 2 – *H*, 0<*H*<1; see Bullmore et al (2004)[11]for review. As *H* approaches 1 the data become increasingly persistent and autocorrelated (long memory dynamics). This 1/*f* power relationship implies that perturbations occurring at slow frequencies can cause a cascade of energy dissipation at higher frequencies so that widespread slow oscillations, observed with BOLD contrast fMRI, are modulating faster local events [12]. In this experiment, values of *H* were decreased during the immediate post-task period with respect to the pre-task values, indicating a relative increase in spectral complexity in the fMRI signal, with gradual recovery to pre-task values over several minutes at a differential rate depending on the load of the preceding cognitive demands.

A previous and innovative fMRI paradigm by Duff et al 2008 [4] found widespread cortical changes in fluctuations at high frequencies in resting state data collected after a finger tapping task, and that this increase in power at high frequencies had not diminished to baseline levels at 4 min post task (as limited by their data acquisition). We extended our data collection to 18 minutes post task and show that in fact it may take up to 15 minutes for the power spectrum (as characterised by the Hurst exponent) to normalise depending on the cognitive load of the preceding task. It is of course often argued endogenous fMRI dynamics, and by implication any changes observed in the BOLD signal activity following task completion, could be associated with the cardiovascular and respiratory processes that form a major component of fMRI signal variation in higher frequency bands [13]. However, this interpretation seems difficult to reconcile with the different rates of time-related change in Hurst exponent of the resting state dynamics measured after tasks which differed only in terms of their cognitive difficulty. A more parsimonious explanation is that post-task changes in endogenous dynamics are directly related to difficulty of task performance.

If it is true that the changes we observe in the Hurst exponent are due to an injection of high frequencies, presumably due to previous task associated neural activity, it could be that the recovery rate in fractal scaling may reflect the brain's ‘journey’ back to some default temporal mode or “state of criticality” that may have some individual cognitive or neurophysiological significance. Fractal or power law scaling, although not definitive of a self organised critical state has been widely accepted as a typical indicator of such a system and the idea of the normal brain existing in a state of criticality fits well with observed normal behaviour: rapid adaptation to minimal inputs, optimized for information transmission, metastable global states, enabling a high capacity for information storage [14]. If this is the case then the metrics of scale invariant systems e.g Hurst exponent, may provide the means to explore the theoretical connection between critical brain dynamics and the adaptivity or variability of human behaviour. However, we could find no relationship between individual levels of performance and fractal dynamics in this study.

Prior studies of endogenous oscillations in EEG and BOLD fMRI have shown a relationship between motor responses and the variability of these signals immediately prior to the motor cue [15], [16]. Compared to the study reported here the time-scales involved in these experiments were much shorter (that is, seconds rather than minutes) and behavioural measures may be thus more closely related to instantaneous signal measures rather than monofractal dynamics that summarize signal complexity over an extended period of time.

Interestingly Duff et al 2008 [4], also found increases in low-frequency spectral power post-task but localised to task-activated regions and suggest that this may reflect persistent intrinsic neural adaptation, or activity associated with consolidation of the practiced motor skill. Alternatively it could simply be indicating the level of drowsiness of the participants during scanning sessions since low frequency changes have been associated with sleep [17]. All participants in our study performed the task successfully and reported being fully awake immediately on completion of this demanding task and none fell asleep during the post-task period. The Hurst exponent, unlike the power spectral density analysis (PSD) used by Duff et al 2008 [4], does not provide information regarding changes in specific frequency ranges and therefore we cannot be sure that the data in our experiment doesn't also show evidence of differential increases in power across particular bandwidths. However, it does provide a powerful summary statistic of how the entire spectrum changes over time due to exogenous stimuli. The combination of data from Duff et al (2008) [4] and the dynamics in the Hurst exponent post-task shown in our data might indicate a possible interaction of scale-free processes with specific high frequency task-associated processes, suggesting that they may originate from different sources.

It will clearly require further studies to establish the generality of this post-task relaxation phenomenon across other tasks; to explore the implications for task performance and related activation if a second task is imposed before recovery from the first task is complete; and to test the hypothesis that abnormal post-task recovery rates might provide a novel marker for neuropsychiatric disorders. However, one immediate general implication is that we should remain thoughtful about the core assumption of reflexive brain function currently central to the conduct and analysis of most human fMRI experiments. These data demonstrate the potential frailty of this approach, or at least indicate that careful attention is needed with regard to task counterbalancing, and when that is not possible (for example, in related encode-retrieve paradigms that are necessarily ordered), account is taken of potential bias in response estimation. Additionally, investigators interested in resting state dynamics should be mindful of the effects that preceding tasks may superimpose upon their data.

## Methods

### Participants

Nineteen right-handed, healthy participants took part in the study. Data from 5 participants were omitted for technical reasons (2 participants failed to follow instructions and 3 sets of behavioural data failed to be recorded) leaving 14 participants for analysis: 8 males; mean age = 23.13 years, range = 21–29 years. All participants were screened for medications, recreational drug use, history of head injury or loss of consciousness, history of prior psychiatric disorder or contraindication to MRI. Participants were screened radiologically for brain structural lesions.

### Ethics Statement

All participants gave informed consent in writing. The protocol was approved by the Addenbrooke's NHS Trust Local Research Ethics Committee (Principal Investigator: E. T. Bullmore; Protocol No. 02/399).

### Study design

Participants were scanned using functional MRI (fMRI) in two separate sessions scheduled at least 30 mins apart. During each session 37 mins 32 s of BOLD contrast data at sampling rate of 1100 ms were continuously acquired using echo-planar imaging (EPI). During the acquisition a period of 9 mins 23 s (512 volumes) of rest (no-task) was followed by the working memory task of 9 mins 23 s (512 volumes), at either a low (one-back) or high (two-back) load, in counterbalanced order across participants. Following the task there was a second period of rest lasting 18 mins 46 s (1024 volumes) (see figure 1). Between imaging sessions participants were asked to sit quietly and not to consume caffeine. The wait between sessions was in the range: 28 mins to 1 hour 32 mins. Most (9/14) participants were scanned within a 30 minute window (45 mins to 1 hour 15 mins). The mean between session time for participants undertaking the 1-back task first (7 participants) was 1 hour 4 mins and the mean between session time for participants undertaking the 2-back task first (7 participants) was 49 mins. There was no significant difference between these mean waiting times (t(12) = 1.533, p = 0.146).

A detailed description of the working memory task is given by Callicot et al [6]. Each visual stimulus was both probe and target and consisted of a sequence of single numbers between 1 and 4 appearing every 1800 ms for 500 ms at set locations at the points of a diamond-shaped box. Instructions displayed above the diamond informed participants to recall the stimulus seen *N* presentations previously. This was done by pressing the button corresponding to the location of the appropriate number using a 4-button box with the same configuration as the stimuli presented on the screen.

The task was presented in a blocked-periodic design with 16×30 s blocks of alternating zero-back trials (that is, locate the current probe on the screen) and either one-back or two-back trials, depending on the session. Accuracy (percent correct) and reaction time (RT) of all responses was recorded. All participants had a 15 minute training session approximately 45 minutes prior to their first session.

During the resting periods prior to and following the working memory task, participants were instructed to lie quietly awake with their eyes closed. At the end of the first rest period two electronic tones alerted participants to open their eyes followed by a 10 s count down to the beginning of the task. A visual instruction indicated that the task had ended.

### FMRI data acquisition

Data depicting BOLD contrast were acquired on a Siemens Tim Trio scanner (http://www.medical.siemens.com/) operating at 3T in the Wolfson Brain Imaging Centre, University of Cambridge, UK. For each EPI acquisition 21 slices of data parallel to the inter-commissural (AC-PC) line were specified with the following parameters: echo time, TE = 30 ms, repetition time, TR = 1100 ms, flip angle = 65°, slice thickness = 4 mm plus 1 mm inter-slice gap, in-plane resolution = 3.0 mm. After discarding 12 initial scans to account for T1 magnetisation effects, each fMRI session acquired 2048 volumes in total: pre-task resting acquisition: 512 EPI volumes; working memory task: 512 EPI volumes; post-task rest acquisition: 1024 EPI volumes.

Structural MRI data, for neuroradiological screening, were also acquired from each participant at the start of the first session: a T1-weighted sequence (voxel size = 1×1×1 mm^3^, field-of-view = 240×256, TI = 900 ms, TE = 2.98 ms, flip angle = 9°) and a dual echo PD/T2 weighted sequence (voxel size = 0.7×0.7×5 mm^3^, FoV = 240×320, TE1 = 11 ms, TE2 = 101 ms, flip angle = 150°).

### Data analysis

Behavioural data were analysed in SPSS (SPSS Inc. version 14). FMRI data were processed and analysed using the Cambridge Brain Analysis (CamBA) analysis suite (http://www-bmu.psychiatry.cam.ac.uk/software) version 1.3.2. Subsequent statistical testing on regional data was performed with SPSS.

Following correction for participant motion, a regression of the general linear model estimated the contrast of working memory active task blocks (either one-back or two-back) versus zero-back blocks. Maps of the estimated responses divided by their standard errors, *F*, were registered into the standard space of the Montreal Neurological Institute (MNI) by an affine transformation to the ‘EPI’ template (http://www.fil.ion.ucl.ac.uk/spm). Within-group median responses of both versions of the working memory task combined were statistically tested against the two-tailed null-hypothesis of no stimulus related activation based on permutation of the original time-series [7], [18]. Probabilistic thresholds at the cluster level were set such that the expected number of false positive clusters was less than one under the null hypothesis. Briefly, maps of *F* statistics under the null-hypothesis of no task activation were estimated from time-series following wavelet permutation and mapped into standard MNI space. Probabilistic thresholding was performed in two-stages: First at the voxel-level, all values at all intracerebral voxels from the permuted response maps were pooled to sample the null-distribution. Voxels with values less than the critical value at p<0.05 were set to zero, whilst those exceeding the critical value were shrunk towards zero by subtracting the critical value. This procedure resulted in sets of three-dimensional voxel clusters in the observed and permuted response maps. Cluster-level statistics were then computed as the sum of suprathreshold voxel statistics for all clusters in all maps. Statistical thresholding at the cluster-level proceeded by pooling cluster statistics from permuted response maps to sample the appropriate null-distribution. Critical values for cluster-level statistics were calculated such that the number of type I errors expected under the null-hypothesis<1. The corresponding equivalent p-value is given.

FMRI datasets acquired during rest were sub-divided into contiguous samples each of 128 time-points. For each sample, a map of the Hurst exponent was estimated for each participant by maximum likelihood in the wavelet domain [2] and registered into MNI standard space by affine transformation. Within the regions significantly activated and deactivated by the working memory task, mean values of *H* were obtained for each participant. Each of 9 longitudinal estimates of the mean regional value of *H* (1 pre-task and 8 post-task), was normalized by subtraction of the estimate from the sample immediately prior to start of the working memory task, to estimate the time-related change in Hurst exponent, Δ*H*.

A repeated measures two-way factorial analysis with factors of working memory load (one-back *versus* two-back), and time (8 samples post-task) was performed with estimates of regional mean Δ*H* as the dependent variable. Sphericity was assumed based on non-significant values of Mauchy's test for each main effect and interaction.
